# Feasibility of an AI-Enabled Smart Mirror Integrating MA-rPPG, Facial Affect, and Conversational Guidance in Realtime

**DOI:** 10.3390/s25185831

**Published:** 2025-09-18

**Authors:** Mohammad Afif Kasno, Jin-Woo Jung

**Affiliations:** 1 Department of Computer Science and Artificial Intelligence, College of Advanced Convergence Engineering, Dongguk University, Seoul 04620, Republic of Korea; mohammad.afif@utem.edu.my; 2 Faculty of Electrical Technology and Engineering, Universiti Teknikal Malaysia Melaka, Melaka 76100, Malaysia

**Keywords:** smart mirror, remote photoplethysmography (rPPG), facial emotion recognition, mental health chatbot, real-time health monitoring, ambient assisted living (AAL), human-centered AI

## Abstract

This paper presents a real-time smart mirror system combining multiple AI modules for multimodal health monitoring. The proposed platform integrates three core components: facial expression analysis, remote photoplethysmography (rPPG), and conversational AI. A key innovation lies in transforming the Moving Average rPPG (MA-rPPG) model—originally developed for offline batch processing—into a real-time, continuously streaming setup, enabling seamless heart rate and peripheral oxygen saturation (SpO_2_) monitoring using standard webcams. The system also incorporates the DeepFace facial analysis library for live emotion, age detection, and a Generative Pre-trained Transformer 4o (GPT-4o)-based mental health chatbot with bilingual (English/Korean) support and voice synthesis. Embedded into a touchscreen mirror with Graphical User Interface (GUI), this solution delivers ambient, low-interruption interaction and real-time user feedback. By unifying these AI modules within an interactive smart mirror, our findings demonstrate the feasibility of integrating multimodal sensing (rPPG, affect detection) and conversational AI into a real-time smart mirror platform. This system is presented as a feasibility-stage prototype to promote real-time health awareness and empathetic feedback. The physiological validation was limited to a single subject, and the user evaluation constituted only a small formative assessment; therefore, results should be interpreted strictly as preliminary feasibility evidence. The system is not intended to provide clinical diagnosis or generalizable accuracy at this stage.

## 1. Introduction

The convergence of healthcare innovation and ambient intelligent technologies has opened new frontiers for non-invasive, contactless, and user-friendly health monitoring solutions. Among these, smart mirror systems—reflective displays augmented with embedded sensors, computer vision, and AI algorithms—have emerged as powerful tools for enhancing personal wellness and remote care environments. As global health systems face rising demands for preventive care, aging population management, and mental health support, smart mirrors provide a unique platform that combines real-time feedback, intuitive interaction, and multimodal sensing without altering daily routines [1,2,3,4,5,6,7,8].

At the core of modern smart mirrors lies a suite of contactless health sensing technologies, including remote photoplethysmography (rPPG) for vital sign monitoring [9], facial expression recognition for affective analysis [3], and AI-driven chatbots for conversational engagement [4]. These tools allow mirrors to unobtrusively assess physical and psychological states, providing users and caregivers with actionable insights. Unlike wearable devices, smart mirrors integrate into daily living spaces and eliminate the need for body attachments, promoting sustained use and better adherence in non-clinical settings [10].

Recent developments in deep learning-based remote photoplethysmography (rPPG) methods, particularly motion-resilient models such as Moving Average remote photoplethysmography (MA-rPPG), have significantly improved the robustness of heart rate and blood oxygen saturation (SpO_2_) estimation from standard webcam feeds [11]. Similarly, facial analysis libraries like DeepFace now enable real-time classification of emotional states, helping to detect early signs of stress, anxiety, or depression [12,13,14]. Coupled with conversational AI-driven chatbots, these components form the backbone of personalized mental and physical health assistants.

**Problem Definition.** Despite these advancements, conventional health monitoring solutions—such as wearables or standalone chatbot apps—often suffer from low user compliance, physical discomfort, or fragmented user experience [5,6]. There is a critical need for a non-intrusive, continuous monitoring system that can seamlessly integrate into users’ daily lives [7,8,15], while simultaneously providing physiological assessment and mental health support in real time [3,4]. Our work addresses this unmet need by unifying advanced AI sensing modalities into an interactive real-time smart mirror interface. Recent works, such as Song et al. (2023), have demonstrated the feasibility of deep learning-based recognition in work-like settings using spatio-temporal facial behavior cues, further highlighting the potential of integrating facial affect analysis into practical systems [16].

While various prototypes have explored individual elements—such as rPPG-enabled mirrors or AI-based emotion trackers, there remains a gap in the integration of multimodal sensing and interaction in a unified system [15] and continuous development by researchers to overcome it [17]. This paper addresses that gap by introducing AI multimodal smart mirror platform that combines three key modules: (1) real-time heart rate estimation using MA-rPPG [11]; (2) affective analysis via DeepFace [12]; and (3) a multilingual mental health chatbot powered by GPT-4o, widely used in healthcare chatbot research [18,19,20,21]. By embedding these technologies into a single cohesive platform, it becomes feasibility prototype to promote real-time health awareness and empathetic feedback.

Our contributions are threefold:

1. **Technical Integration**: We design and implement a modular smart mirror system incorporating validated tools for real-time physiological and emotional sensing.
2. **Real-Time Interaction**: Our platform supports AI Multimodal interaction for low-latency, bidirectional communication in both English and Korean languages, enabling interactive Smart Mirror.
3. **Online MA-rPPG Innovation**: While prior MA-rPPG implementations function in offline mode using pre-recorded videos, we adapt it for real-time inference through continuous webcam streaming and GPU acceleration.

**Figure 1:**
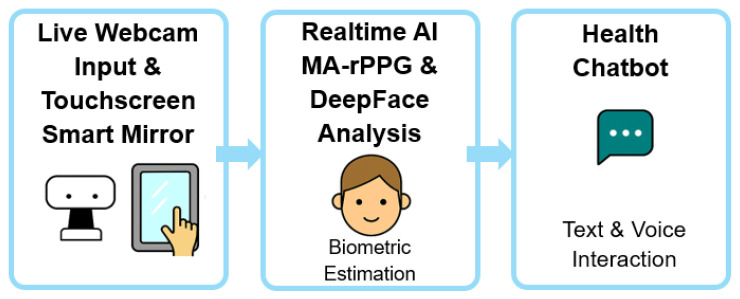
Graphical Abstract of proposed methodology.

The rest of this paper is organized as follows: Section 2 reviews related work in rPPG sensing, affective computing, chatbot-assisted mental health, and smart mirror platforms. Section 3 details the system architecture and implementation, while Section 4 and Section 5 cover performance evaluation and discussion, respectively. The graphical abstract of proposed methodology is presented as Figure 1.

## 2. Related Works

### 2.1. Remote Photoplethysmography (rPPG) for Ambient Health Monitoring

Remote photoplethysmography (rPPG) has emerged as a critical modality for contactless health monitoring, enabling real-time measurement of physiological parameters such as heart rate and blood oxygen saturation without direct skin contact. Unlike traditional photoplethysmography (PPG) that requires sensor placement on the skin, rPPG leverages standard RGB video feeds to detect pulse-induced color fluctuations across facial regions. This modality is particularly suited for integration into ambient systems like smart mirrors due to its non-intrusive nature and compatibility with consumer-grade hardware [22,23,24,25,26,27,28].

Despite its growing adoption, the development of reliable and real-time rPPG systems remains technically challenging due to motion artifacts, lighting inconsistencies, and signal-to-noise ratio limitations. Recent studies underscore the importance of modular and explainable pipelines, where individual components—such as Region of Interest (ROI) detection, motion compensation, and frequency tracking—are optimized and evaluated independently to improve robustness in dynamic settings like fitness environments [22]. Moreover, statistical evaluations across public datasets reveal inconsistencies in performance across methods such as POS, CHROM, PCA, and LGI, emphasizing the need for standardized benchmarking frameworks like pyVHR [27]. Some works, like those by Gudi et al., propose real-time, CPU-efficient pipelines that prioritize heartbeat-level precision to enable HRV estimation, moving beyond average heart rate metrics [25]. Others leverage deep learning or machine learning techniques to reconstruct cleaner rPPG waveforms from noisy RGB input, improving temporal alignment and physiological fidelity in diverse contexts [26]. However, even these advanced systems must balance model complexity with runtime constraints to ensure seamless integration in edge devices like smart mirrors.

Recent advancements in rPPG methodologies address several challenges such as motion artifacts, lighting variation, and skin tone diversity. Casalino et al. developed a low-cost smart mirror platform for rPPG-based monitoring, demonstrating improved robustness to motion and real-time usability in domestic environments by refining the video processing pipeline and implementing enhanced signal stabilization algorithms [9]. Complementing this, Paruchuri et al. introduced MA-rPPG—a neural motion transfer-based data augmentation technique—that significantly improves inter-dataset generalization by simulating naturalistic head movements, thereby enhancing model robustness under diverse real-world scenarios [11]. It is interesting to further improve MA-rPPG from offline into real-time implementation through our AI Multimodal Smart Mirror development.

### 2.2. Deep Learning-Based Facial Emotion Recognition for Mental Health Monitoring

A recent comprehensive survey conducted by Canal et al. (2022) systematically reviewed the state-of-the-art techniques in facial emotion recognition (FER), categorizing methods primarily into classical image processing-based techniques and neural network-based approaches [29]. Their analysis highlighted the increasing prominence of Convolutional Neural Networks (CNNs) due to their superior capacity to autonomously learn discriminative facial features, outperforming classical methods in generalization to diverse datasets and real-world conditions. Furthermore, the survey emphasizes the critical importance of robust and diverse datasets, pointing out limitations in current benchmark databases that constrain the real-world applicability of FER systems. This detailed review underscores not only the technical advancements in deep learning architectures but also sheds light on persistent challenges such as cross-dataset generalization, environmental variability, and the necessity for more inclusive and representative datasets to enhance the clinical applicability of FER for mental health monitoring and intervention [29,30,31,32,33,34,35].

Hybrid CNN-LSTM architectures have been shown to improve continuous emotion recognition, with studies reporting performance gains exceeding 12% over static image-based CNNs [30]. Benchmark models fine-tuned on standard datasets like JAFFE [31] and KDEF [32] have achieved classification accuracies of 96–99%, confirming the viability of real-time deployment in mental health monitoring systems [33]. Libraries such as DeepFace integrate pre-trained deep embeddings with multi-detector pipelines, supporting high-resolution, real-time emotion tracking on edge devices such as smart mirrors [12].

Clinically, emotion recognition systems are increasingly applied in monitoring affective states associated with depression, anxiety, and mood disorders. By continuously analyzing micro-expressions and temporal emotion patterns, these systems provide an objective framework for early detection and intervention. Notably, FER has also demonstrated utility in pediatric applications, including autism spectrum disorder (ASD) diagnosis, by enabling the identification of atypical affective responses [29,30,34]. Song et al. (2023) further extended this research to work-like environments, proposing a novel dataset and benchmarking framework for affect recognition based on spatio-temporal facial behaviors, providing insights into real-world deployment considerations [16].

Within developed smart mirror, emotion recognition functions as both a diagnostic and interactive component, enabling adaptive interfaces that tailor feedback and recommendations to the user’s emotional state in real-time.

### 2.3. Conversational AI for Mental Health Interventions

Conversational artificial intelligence (AI) systems are increasingly utilized in healthcare to provide scalable, real-time, and user-centric interventions. In the realm of mental health, AI-powered chatbots have emerged as virtual companions capable of delivering cognitive-behavioral therapy (CBT), mood tracking, and psychoeducation through natural language interfaces. These digital agents offer immediate, round-the-clock support, enhancing accessibility to mental health resources, especially for individuals who may face barriers to traditional therapy [36,37,38]. Studies have demonstrated the efficacy of such interventions; for instance, Woebot, a fully automated conversational agent, has been shown to significantly reduce symptoms of depression and anxiety within two weeks of use [39].

Modern conversational AI systems leverage transformer-based language models that utilize contextual embeddings for dynamic dialog generation. By fine-tuning these models on healthcare-specific corpora, they can perform tasks such as symptom triage, mood inference, and delivering empathetic responses. For example, the CBT-LLM model, a large language model tailored for cognitive behavioral therapy, has demonstrated proficiency in generating structured and professional responses in psychological health support tasks. These advancements have the potential to enhance patient engagement, reduce anxiety through cognitive reframing, and serve as interim mental health support, particularly during periods of provider shortages [40].

However, the deployment of conversational AI in sensitive contexts like mental health raises significant equity and ethical concerns. Ensuring fairness, avoiding algorithmic bias, and maintaining privacy in emotion-sensitive interactions are critical for trustworthy system design. A comprehensive overview of ethical considerations highlights issues such as the need for transparency, informed consent, and the inclusion of diverse perspectives during model training and validation phases. Addressing these concerns is essential to prevent unintended harm and to build user trust in AI-driven mental health interventions [41].

In the context of smart mirrors, conversational AI transforms the reflective surface into a responsive interface—offering daily check-ins, mood assessments, and therapeutic dialogs. Integrated with rPPG and FER inputs, chatbot systems can personalize interventions based on detected physiological and emotional cues, providing a multimodal framework for comprehensive mental wellness support [42].

### 2.4. Integration in Smart Mirror Healthcare Systems

The integration of rPPG, emotion recognition, and conversational AI into smart mirror platforms marks a significant advancement in ambient health intelligence. These systems redefine personal health monitoring by enabling seamless, contactless interaction without disrupting user routines. Smart mirrors, typically composed of a two-way acrylic display, embedded camera, microphone, and edge computing unit, provide an ideal medium for daily physiological and psychological assessments.

The system architecture typically follows a modular pipeline: the webcam captures facial videos, which are simultaneously processed for vital sign estimation via rPPG (e.g., MA-rPPG) and emotion analysis using facial recognition libraries (e.g., DeepFace). Speech input is processed via natural language understanding modules linked to AI-powered chatbots (e.g., GPT-4o), generating real-time conversational feedback. This multi-stream pipeline is orchestrated through RESTful Application Programming Interfaces (APIs) and rendered via GUI layers on React-based frontends.

Previous implementations, such as the mirror by Casalino et al. [9], focused on physiological monitoring but lacked affective computing integration. The proposed system extends these efforts by unifying biometric sensing and AI interaction within a bilingual, privacy-aware interface, tested under real-world ambient conditions. By leveraging the MA-rPPG model’s robustness to motion and the conversational depth of modern language models, the mirror adapts dynamically to user states, offering personalized feedback across mental and physical dimensions.

This synthesis of technologies supports both preventive health measures and early intervention strategies, aligning with the broader vision of ambient assisted living and patient-centered digital therapeutics.

## 3. System Development Overview

In this work, our innovation lies in the integration of multiple validated AI models into a real-time smart mirror system rather than the development of new algorithms. We selected MA-rPPG, DeepFace, and GPT-4o based on their documented performance in prior studies. Paruchuri et al. [11] demonstrated that MA-rPPG significantly improves inter-dataset generalization and reduces heart rate estimation error. DeepFace, as benchmarked by Serengil et al. [12], achieves 96–99% classification accuracy on standard emotion datasets. GPT-4o, described in its technical documentation and recent studies, has shown state-of-the-art performance in natural language generation for healthcare applications. These validated models provide robust components for our integration-focused development.

The smart mirror system is developed as a modular and event-driven architecture, integrating multiple components into a cohesive real-time pipeline. The system begins with a hardware layer that captures live biometric signals through a standard webcam and a USB microphone. These signals are processed on a GPU-enabled edge device running an Ubuntu 20.04-based system with an NVIDIA RTX 4060 Ti GPU to support deep learning inference at high frame rates.

The backend processing layer consists of two main Flask-based microservices. One is to handles real-time rPPG signal processing using the MA-rPPG model for heart rate and SpO_2_ estimation and concurrently extracts facial attributes—age and emotion—via DeepFace. Simultaneously, the second one manages AI-driven conversational interaction by converting voice input to text, performing mental wellness prompt generation using GPT-4o, and rendering synthesized speech using OpenAI’s Nova voice.

The application interface layer is implemented using React.js and provides dynamic communication with the backend via RESTful endpoints. Core modules manage state control, API polling, and user interface rendering. Once the system validates the presence of meaningful biometric and affective data, it triggers prompt generation and dispatches it to the chatbot server.

**Figure 2:**
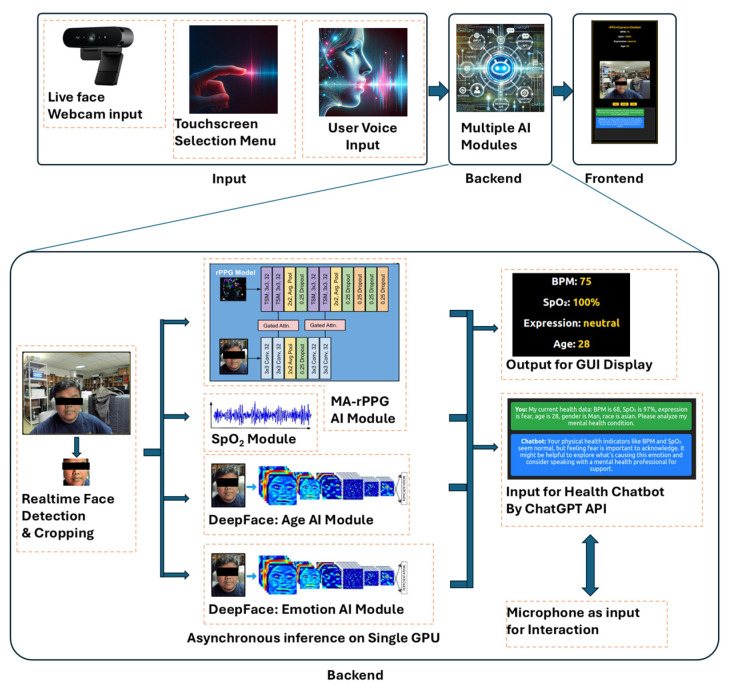
Smart Mirror System Workflow: MA-rPPG, DeepFace, and Chatbot Integration.

Finally, the Graphical User Interface (GUI) is rendered on a 24-inch touchscreen monitor overlaid with a two-way acrylic mirror. The GUI displays real-time vital signs, demographic information, emotional expression, and chatbot responses in both textual and audio form. The entire development workflow depicted in the Figure 2 demonstrates a complete loop—from sensor data acquisition to intelligent feedback delivery—offering a robust, low-latency, and human-centric interaction paradigm for ambient health monitoring.

### 3.1. Hardware Setup

**Figure 3:**
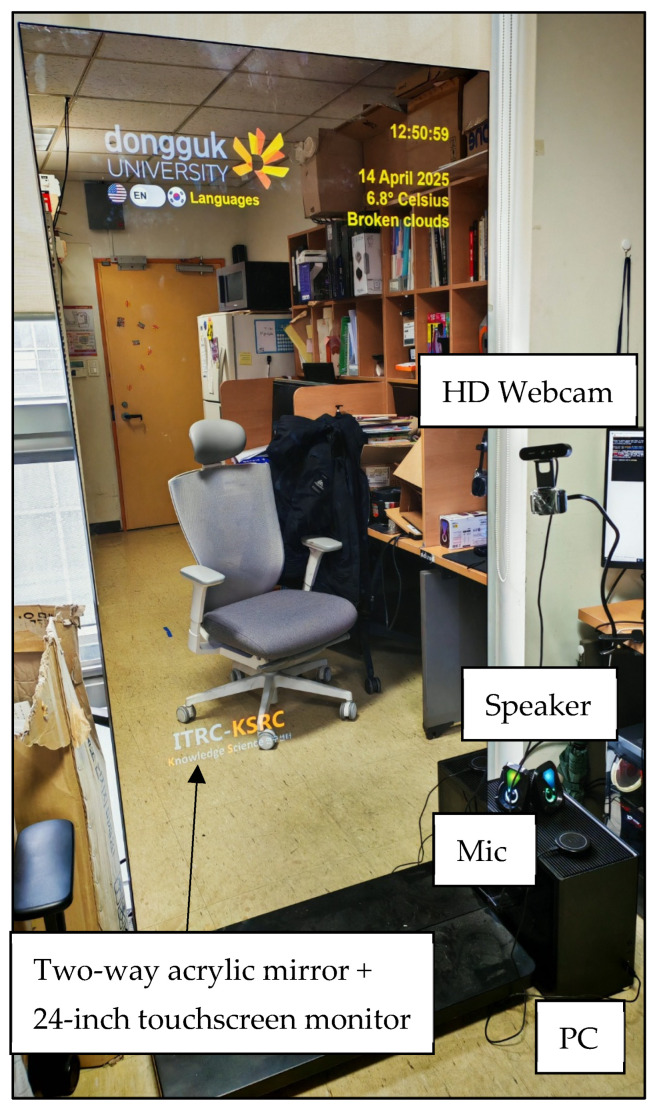
Hardware Setup of Smart Mirror.

The smart mirror prototype is constructed using a two-way acrylic mirror mounted onto a 24-inch touchscreen monitor (Toshiba Corporation, Tokyo, Japan), serving both as a reflective surface and a graphical display unit. The display is connected to a high-performance edge computing system consisting of a desktop workstation running Ubuntu 20.04 LTS and equipped with an NVIDIA RTX 4060 Ti GPU (NVIDIA Corporation, Santa Clara, CA, USA). This hardware configuration is optimized for deep learning inference, enabling real-time processing of high-resolution video data and low-latency rendering, see Figure 3.

A standard HD webcam MISP-REM-DZL-V-0004 (Logitech International S.A., Lausanne, Switzerland) is positioned at eye level to continuously capture facial imagery for physiological signal extraction and emotion recognition. The webcam’s output serves as the input stream for the MA-rPPG-based heart rate and SpO_2_ estimation module as well as the DeepFace-powered facial analytics pipeline. In addition, a ReSpeaker USB microphone and speaker array (Seeed Technology Co., Ltd., Shenzhen, China) facilitates seamless voice interaction, supporting both speech-to-text (via Google Speech API) and text-to-speech (via OpenAI Nova voice model) processes integrated into the GPT-4o chatbot system.

This hardware arrangement allows the smart mirror to support continuous multimodal input-output cycles while maintaining high visual fidelity and interactive performance. The complete setup is deployed within a modular, vertically mounted enclosure and placed in a real-world office environment to simulate ambient noise and lighting variability, enhancing its practical relevance and robustness.

### 3.2. Integrated Hardware-to-GUI Pipeline Architecture

**Figure 4:**
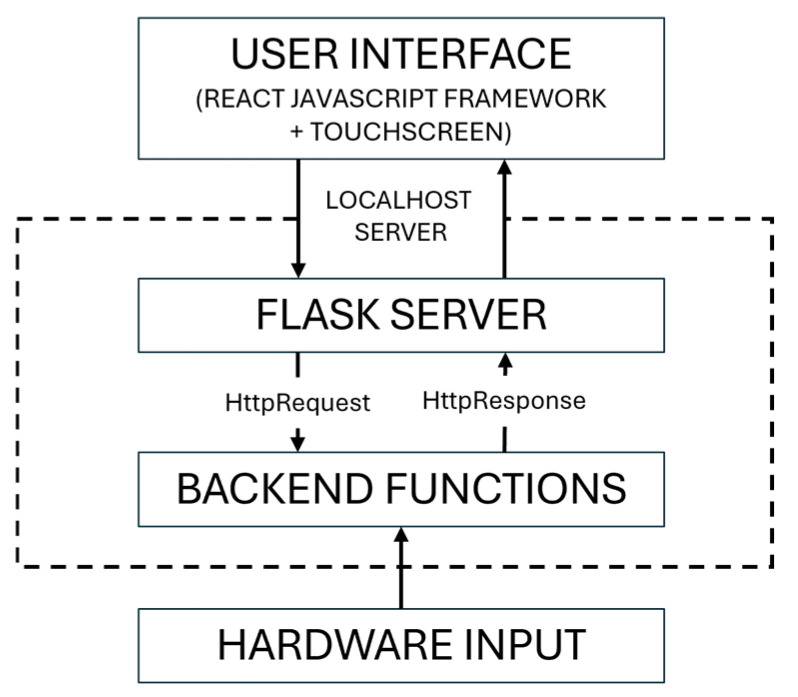
Data Exchange Architecture between Frontend and Backend Modules in Smart Mirror System.

The Figure 4 illustrates the full-stack architecture of the smart mirror system, highlighting the logical flow from sensor input to intelligent user feedback. The system begins at the hardware level, where a webcam and microphone continuously capture video and audio signals. These are processed in real time by a GPU-enabled local server.

The backend layer is composed of modular microservices that handle physiological signal extraction and natural language interaction. One module estimates vital signs and analyzes facial expressions and demographic features, while another module manages user dialog—interpreting spoken input, generating responses using a language model, and converting text back into speech.

These backend services communicate with the frontend via secure web-based interfaces. The graphical interface, implemented as a responsive application, regularly retrieves updated biometric and conversational data to drive the user experience. It orchestrates user interaction, visual updates, and feedback cycles through asynchronous data flow.

Finally, the smart mirror display presents real-time health insights, emotional state summaries, and verbal or textual feedback in a clean and bilingual user interface. The design prioritizes accessibility and ambient integration, making it suitable for continuous wellness monitoring in everyday environments.

### 3.3. Smart Mirror Software Architecture

#### 3.3.1. Smart Mirror Main GUI

**Figure 5:**
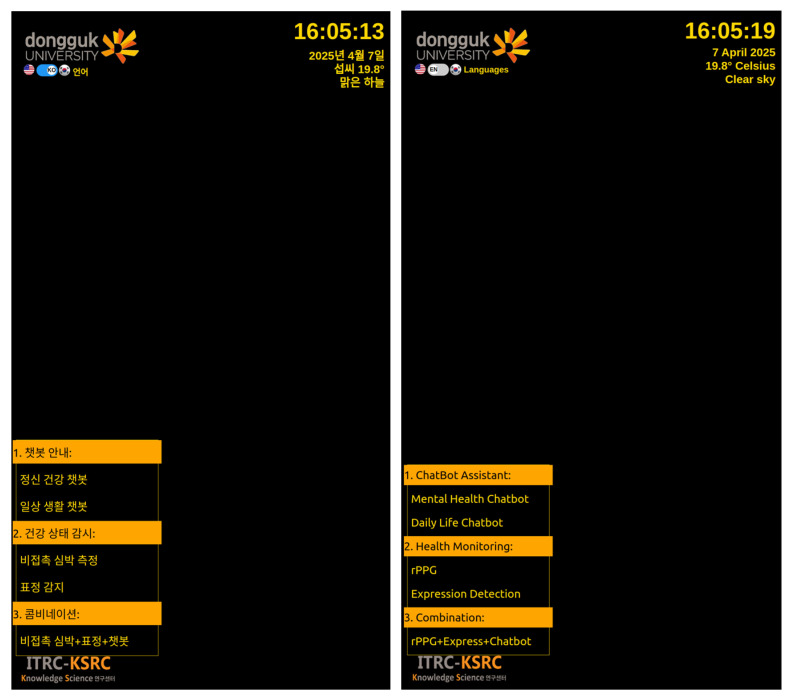
The Main Graphical User Interface (GUI) of Smart Mirror. The interface supports both Korean and English language modes; therefore, the figure illustrates the same GUI displayed side by side in Korean (left) and English (right).

The main graphical user interface (GUI) of the smart mirror is designed as a real-time, bilingual digital dashboard that facilitates intuitive interaction for health monitoring, mental wellness support, and environmental awareness. The interface follows a minimalist and accessible design approach, ensuring clarity and ease of use for a wide range of users, including older adults and individuals with limited technical experience, see Figure 5.

Displayed on a 24-inch touchscreen embedded behind a two-way acrylic mirror, the GUI presents essential information such as current time, date, local weather updates, and system status. Users interact with the system through a structured menu organized into three primary service categories: (1) a conversational assistant for mental health and daily communication; (2) a health monitoring module for real-time analysis of physiological and emotional states; and (3) a combined service mode that synchronizes multiple sensing features for empathetic chatbot feedback.

The interface supports both Korean and English, allowing users to switch languages instantly based on preference. Upon interaction, the GUI triggers backend processes that handle biometric sensing, emotional state analysis, and natural language conversation, returning results dynamically to the screen and, where applicable, through voice feedback. Each function operates in real time, enabling the system to adapt to the user’s current state and deliver personalized feedback without interruption.

Overall, the GUI serves as the central layer that unifies sensory data collection, AI-driven inference, and user interaction into a seamless ambient experience—designed not only to monitor but also to enhance the user’s daily wellbeing through subtle, non-intrusive engagement.

#### 3.3.2. Integrated Multimodal Feedback Submodule

**Figure 6:**
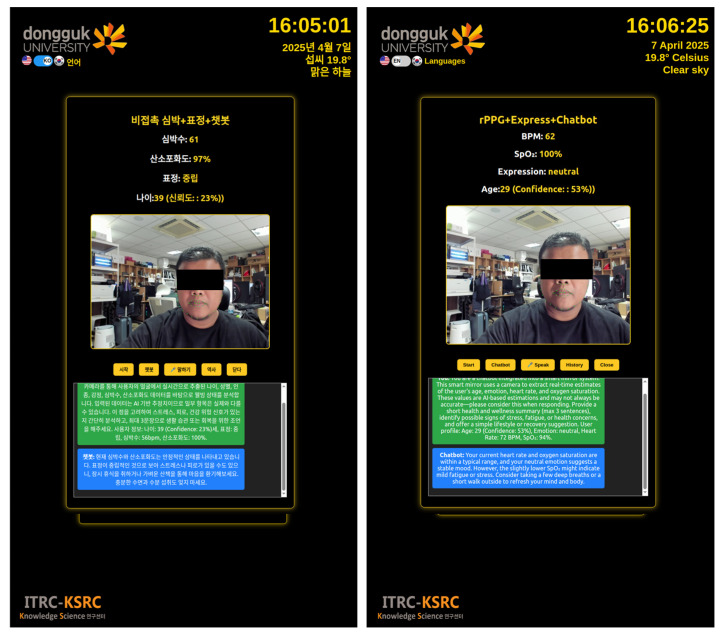
The Combination Submodule. The interface supports dual language (Korean on the left, English on the right) showing realtime BPM, SpO_2_, Expression and Age estimation.

This submodule integrates three key components—physiological monitoring, facial expression analysis, and conversational AI—into a unified, real-time interaction pipeline. The system continuously analyzes live video input to estimate heart rate, oxygen saturation, emotional state, and demographic attributes. These multimodal data points are then synthesized to generate personalized health feedback and wellness summaries in real time. At present, the feedback is provided only for immediate user interaction and is not stored. In future development, we plan to incorporate a user profiling module to maintain a historical record for longitudinal monitoring and more personalized recommendations, see Figure 6.

Once sufficient biometric and affective data are gathered, the system triggers an intelligent response mechanism. A virtual assistant processes the user’s current physiological and emotional context and delivers brief, empathetic guidance through both visual display and spoken output. This interaction is dynamically adjusted based on the user’s current state, providing a seamless blend of sensing and feedback.

The entire cycle operates in real time, enabling synchronized updates between physiological signal processing, emotion recognition, and dialog generation. This tightly integrated interaction model allows users to receive timely, relevant insights without the need for manual input or switching between modes.

By combining contactless sensing and AI-driven reflection into a single experience, this submodule enhances user engagement and enables a more natural and continuous approach to self-awareness, stress detection, and mental health support.

#### 3.3.3. Blood Oxygen Saturation (SpO_2_) Estimation

The estimation of blood oxygen saturation (SpO_2_) in the Smart Mirror system is based on chrominance signal analysis from facial video streams, as described by Kong et al. [40]. It is then operationalized in the rPPG-new methodology outlined by Casalino et al. [9]. This approach leverages the differential absorption properties of human hemoglobin in the red (660 nm) and blue (940 nm) spectral bands to infer oxygenation levels non-invasively from skin reflectance.

From the facial Regions of Interest (ROIs), pixel-wise intensity values across the red (R), green (G), and blue (B) channels are spatially averaged per frame to construct the RGB signal matrix:

(1) $$VR=\frac{1}{nm}\sum_{i=1}^{n}\sum_{j=1}^{m}R_{ij},VG=\frac{1}{nm}\sum_{i=1}^{n}\sum_{j=1}^{m}G_{ij},VB=\frac{1}{nm}\sum_{i=1}^{n}\sum_{j=1}^{m}B_{ij}\;$$

This results in a 3 × N temporal signal matrix VRGB, which captures physiological pulsatility along with environmental noise. To extract the plethysmographic component relevant for oxygenation, a chrominance-based signal separation method is applied, converting RGB signals into orthogonal chrominance traces:

(2) $$X_{s}=3VR-2VG\;$$

(3) $$Y_{s}=1.5VR+VG-1.5VB$$

These signals are further filtered using a Finite Impulse Response (FIR) bandpass filter to attenuate non-physiological frequency components, typically preserving only the [0.6–4 Hz] band. The final blood volume pulse signal *S* is then computed using a skin-tone normalization factor *α*, defined as the ratio of standard deviations:

(4) $$S\;=\;X_{f}-\;\alpha \;Y_{f},\;\alpha \;=\frac{\sigma \left(X_{f}\right)}{\sigma \left(Y_{f}\right)}$$

To derive the SpO_2_ value, signal decomposition is performed to obtain the direct current (DC) and alternating current (AC) components from the red and blue channels of the VRGB signal:

(5) $$DC_{RED}=\;\mu \left(R\right),\;AC_{RED}=\;\sigma \left(R\right),\;DC_{BLUE}=\;\mu \left(B\right),\;AC_{BLUE}=\;\sigma \left(B\right)$$

These parameters are then used in the empirically derived ratio-of-ratios formula:

(6) $$SpO_{2}=\;A\;-\;B\;*\;\left(\frac{\frac{AC_{RED}}{DC_{RED}}}{\frac{AC_{BLUE}}{DC_{BLUE}}}\right)$$

The coefficients A=125 and B=26 adopted from Kong et al. [43] are used to calibrate the model across varying skin tones and illumination conditions. This formulation allows for frame-level estimation of SpO_2_ using only ambient lighting and a standard RGB camera. The resulting SpO_2_ values showed preliminary similarity to readings from a reference finger pulse oximeter (Qinhuangdao Contec Medical Systems Co., Ltd. Shenzhen, China; typical accuracy ±2%) in controlled conditions during a single-subject test. These outputs should be regarded as prototype-level estimates, not validated clinical measurements. Clinical benchmarking across multiple participants remains future work.

The entire estimation process is conducted over a two-second video window, following a 26 s signal stabilization phase, to enable near-real-time SpO_2_ assessment without requiring user contact or specialized optical hardware.

#### 3.3.4. MA-rPPG Estimation Module

The MA-rPPG model (Motion-Augmented Remote Photoplethysmography) introduces a novel neural pipeline designed to enhance the generalizability and robustness of camera-based physiological measurement by leveraging neural motion transfer for training data augmentation. Traditional rPPG models are vulnerable to noise from rigid and non-rigid facial motions. MA-rPPG mitigates this by synthesizing training data where motion is decoupled from the physiological signal, preserving the true photoplethysmography component while introducing controlled variations in motion [11].

The core of the MA-rPPG framework is the motion augmentation pipeline, which synthesizes new facial videos by transferring realistic head and expression motions from a driving video $D=\left\{d_{1},d_{2},\ldots ,d_{n}\right\}$ to a source video $S=\left\{s_{1},s_{2},\ldots ,s_{n}\right\}$ that contains known physiological ground truth (e.g., PPG waveform). The motion-augmented sequence $Y=\left\{y_{1},y_{2},\ldots ,y_{n}\right\}$ is obtained via a neural motion transfer function $M\left(\cdot ;\theta \right)$ where the inputs are the source and driving video frames, as defined in Equation (7):

(7) $$y_{t}=M\left(s_{t},d_{t};\theta \right)$$

This transformation is achieved using Face-Vid2Vid, a keypoint-based generative model that teaches facial motion dynamics. Keypoints representing facial structure and expression are extracted and matched between the source and driving videos. The algorithm then generates each frame $y_{t}$ such that it preserves the identity and skin reflectance of the source while incorporating motion characteristics from the driver. To verify physiological fidelity, frequency domain analysis confirms that the heart rate peak (dominant frequency) in the synthesized video remains consistent with the source, validating the preservation of the rPPG signal. This is further reinforced by signal-to-noise ratio (SNR) assessments and downstream performance on trained rPPG networks such as TS-CAN, PhysNet, and DeepPhys.

The motion-augmented videos are used to train deep learning models for PPG estimation. These models receive as input the spatio-temporal video frames and learn to regress the first-order derivative of the PPG signal. The loss function is defined as a mean squared error (MSE) between the predicted and ground truth PPG signals:

(8) $$L_{MSE}=\left(\frac{1}{T}\right)\sum_{t=1}^{T}{\left(P{\hat{P}G}_{t}-PPG_{t}\right)}^{2}$$

To derive heart rate (HR), the estimated PPG is transformed into the frequency domain using a Fast Fourier Transform (FFT). The frequency corresponding to the peak spectral power is denoted as $f_{h}$ and the HR is calculated as:

(9) $$HR=f_{h}\times 60\;(bpm)$$

This model has demonstrated state-of-the-art performance in inter-dataset scenarios. Training on motion-augmented datasets has shown to reduce mean absolute error (MAE) in HR estimation by up to 79%, highlighting the effectiveness of this augmentation strategy for achieving robust physiological sensing in unconstrained environments.

#### 3.3.5. Emotion Detection Module

In the proposed smart mirror system, facial emotion recognition is accomplished using the DeepFace library, which operates through a robust four-stage deep learning pipeline [12]. This pipeline comprises: (1) face detection, (2) facial alignment, (3) feature embedding via a convolutional neural network (CNN), and (4) classification using distance-based similarity metrics. Let the input frame from the webcam be denoted as $I\in R^{H\times W\times 3}$ where I represent the image and $R^{H\times W\times 3}$ is a real-valued tensor for height × width × 3 RGB channels. The system processes each frame as follows:

##### Face Detection and Alignment

A face detector D locates the bounding box $B=\left(x,y,w,h\right)$ around the face:

(10) B=D(I)

Facial landmarks ${\left\{\left(x_{i},y_{i}\right)\right\}}_{i=1}^{k}$, particularly the positions of the two eyes, are extracted to compute the alignment angle A. A right triangle is constructed where

a = distance between the two eyes,
b and c = distances from each eye to a reference point (e.g., nose tip or a midpoint below the eyes).

The angle A between the baseline of the eyes and the horizontal axis is then computed using the cosine rule:

(11) $$\cos\left(A\right)=\frac{b^{2}+c^{2}-a^{2}}{2bc}$$

(12) $$A^{\circ }=\arccos\left(\frac{b^{2}+c^{2}-a^{2}}{2bc}\right)\cdot \frac{180}{\pi }$$

In Formula (12), the alignment angle A is computed to horizontally align the detected facial landmarks for robust facial analysis. Parameter a specifically represents the distance between the two eyes, while parameters b and c denote distances from each eye to a selected central reference point, such as the midpoint of the nose bridge. This clarification explicitly defines each parameter’s meaning and ensures that readers clearly understand the geometric relationships essential for consistent facial landmark alignment and subsequent emotion and physiological analyses.

##### Feature Representation via Deep CNN

The aligned face region $I_{B}$ is resized to the input shape required by the CNN Φ. For DeepFace, this is typically 152×152×3. The model generates a high-dimensional embedding $v\in R^{d}$, where d=4096:

(13) $$v=\Phi \left(I_{B}\right)$$

This embedding encodes emotional and demographic information in a compact vector space.

##### Emotion Classification

To classify emotion, DeepFace compares v against labeled prototypes ${\left\{v_{j}\right\}}_{j=1}^{C}$ using cosine similarity:

(14) $$\mathrm{sim}\left(v,v_{j}\right)=\frac{v\cdot v_{j}}{|v{|}_{2}\cdot |v_{j}{|}_{2}}$$

The predicted class y is then:

(15) $$y=arg\underset{j}{\max}sim\left(v,v_{j}\right)\;$$

Alternatively, Euclidean distance may be used:

(16) $$d\left(v,v_{j}\right)=\sqrt{\sum_{i=1}^{d}{\left(v_{i}-v_{j,i}\right)}^{2}}$$

In this case, the predicted class minimizes the distance:

(17) $$y=arg\underset{j}{\min}d\left(v,v_{j}\right)$$

#### 3.3.6. Chatbot Interaction Module

The chatbot interaction module in the smart mirror system is designed to deliver concise, empathetic, and context-aware mental wellness responses by integrating biometric signal processing with natural language generation and speech synthesis. This module operates at the convergence of physiological monitoring, affective computing, and conversational AI. At the back end, a Flask server hosts the chatbot engine, which interfaces with OpenAI’s GPT-4o model (May 2025 release) accessed via RESTful API. The interaction is initiated following a 10 s physiological profiling window, after which biometric sensing modules collect heart rate, oxygen saturation, emotion, and age estimation data. A trigger threshold was set such that chatbot feedback is generated only when at least 8 s of valid rPPG frames (signal-to-noise ratio > 0.7) and a face-detection confidence >70% are achieved. This ensures stable input before initiating conversation. Once sufficient data integrity is validated, the frontend issues a dynamic prompt embedding these multimodal inputs into a structured query.

The system then leverages a dual-stage AI interaction pipeline: first, GPT-4o generates a personalized wellness summary constrained to two or three empathetic sentences; second, the generated text is synthesized into speech using Text-to-Speech (TTS) model. This synthesized audio is streamed to the user for naturalistic interaction. To support multilingual accessibility, the module includes bilingual prompt templates and toggles for Korean and English interfaces. Additionally, voice input is supported using Speech-to-Text (STT), allowing further communication between user and the chatbot regarding the biometric sensing results. This integrated chatbot system exemplifies a contextually intelligent digital assistant, dynamically adapting its responses based on biometric context rather than static user queries.

#### 3.3.7. GUI and Programming Flow

The smart mirror system follows a step-by-step interaction flow, designed to deliver health insights and chatbot feedback in a smooth and timely manner. Once the system is started, the webcam and microphone are activated, and the display interface becomes visible on the smart mirror screen. In the first stage, the system continuously analyzes the user’s face using the built-in camera. It estimates heart rate, blood oxygen saturation (SpO_2_), and facial expression in near real time. At the same time, it captures information such as age estimation from the user’s appearance.

After collecting enough information, the system displays health status and emotional state on the screen. If the user activates the chatbot feature, the system combines the collected data into a single health summary and sends it to the AI chatbot assistant. The chatbot then responds with supportive feedback, which appears on the screen and plays as audio. The user is then able to respond by voice to further communicate with the chatbot regarding feedback.

**Figure 7:**
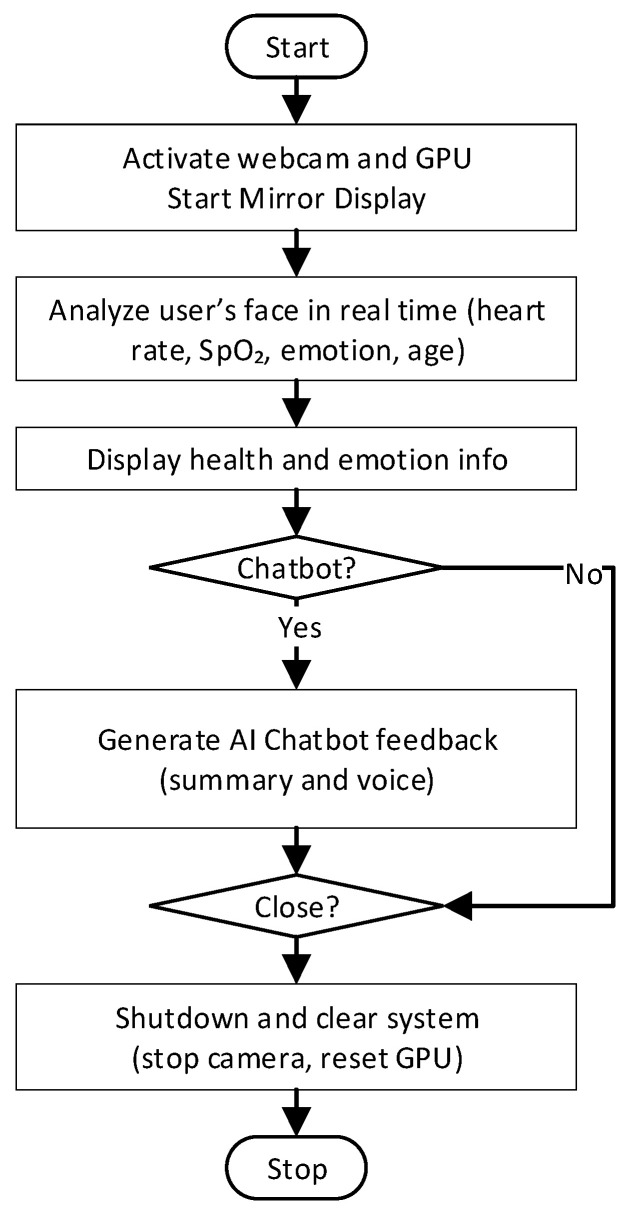
Programming Flowchart of AI Multimodal Smart Mirror.

At any point, the user can choose to stop the system. When this happens, all processes are safely shut down, the webcam and GPU are disengaged, and system memory is cleared. Processing windows were set at 2 s for rPPG/SpO_2_ estimation with a 26–30 s stabilization period. Age classification used rolling confidence sampling (30 iterations, majority voting as final output). Chatbot responses were configured through a constrained prompt template with bilingual support (English/Korean), limited to 2–3 empathetic sentences to minimize latency. Average chatbot API latency was 1200–1800 ms. This step-by-step loop—starting from sensing and ending with personalized feedback—creates a complete interaction cycle that helps users reflect on their health and wellbeing through a simple and natural interface. The flowchart is illustrated in Figure 7 below:

## 4. Experimental Evaluation

To thoroughly evaluate the developed AI multimodal smart mirror system, three detailed experiments were designed and performed. Each experiment addresses a crucial aspect of the system, including computational performance, method accuracy, and multimodal parameter validation. Below, the experimental setups are clearly outlined, with placeholders provided for the forthcoming results.

### 4.1. Evaluation Protocols

To ensure reproducibility, all evaluations followed a standardized experimental protocol:

- Hardware and Camera Specifications: All tests were performed on a desktop workstation (Ubuntu 20.04 LTS, NVIDIA RTX 4060 Ti GPU(NVIDIA Corporation, Santa Clara, CA, USA). A Logitech HD webcam was used at 640 × 480 resolution and 30 frames per second, positioned at eye level.
- Subject Distance and Pose: Participants were seated at a distance of 0.8–1.0 m from the mirror, maintaining a frontal pose without significant head rotation.
- Lighting Conditions: Experiments were conducted under typical office ambient lighting (300–350 lux). No direct sunlight or additional studio lighting was used.
- Stabilization Period: A 30 s stabilization window was applied prior to each measurement for rPPG/SpO_2_ signal normalization, consistent with prior rPPG practices.
- Window Lengths: rPPG and SpO_2_ estimation used 2 s rolling windows for frame-level computation.
- Number of Trials and Subjects: Each subject completed three consecutive trials, and results were averaged.
- Exclusion Criteria: Trials were discarded if the subject moved abruptly, left the camera frame, or if face detection confidence dropped below 70%. Trigger thresholds included: minimum 70% face-detection confidence, minimum 8 s rPPG window with acceptable SNR, and successful extraction of at least one affective label with >50% confidence. These thresholds determined whether data were passed to the chatbot module for response generation.

For affective analysis, participants were instructed to maintain a neutral pose and respond naturally while interacting with the mirror. The DeepFace module was validated by asking participants to display a sequence of basic emotions (happy, sad, neutral, fear, angry), and classification accuracy was computed.

For the chatbot evaluation, participants were instructed to ask two types of queries: (1) wellness-related prompts (e.g., “How am I doing today?”) and (2) open-ended small talk. Each response was assessed based on (a) latency (ms), (b) relevance (whether the response matched the context of biometric/affective cues), and (c) user-perceived empathy, rated on a 5-point Likert scale.

All participants provided informed consent prior to involvement in the formative evaluations. No personal identifiers were recorded, and all biometric processing was conducted locally on the edge device without storage of raw video or audio data, ensuring privacy.

### 4.2. System Performance: Latency and GPU Utilization

To measure the real-time computational efficiency of the multimodal smart mirror system, GPU usage percentage, latency, and chatbot response times were systematically recorded. Tests were conducted using an NVIDIA RTX 4060 Ti GPU, employing a webcam set at a resolution of 640 × 480 pixels and capturing data at 30 frames per second. The measurements for GPU utilization, latency of the rPPG processing, DeepFace expression detection, and chatbot response were collected over five-minute intervals under controlled environmental conditions. The results will be detailed as follows (see Table 1):

### 4.3. Physiological Accuracy: Real-Time MA-Rppg vs. Contact PPG Sensor

**Figure 8:**
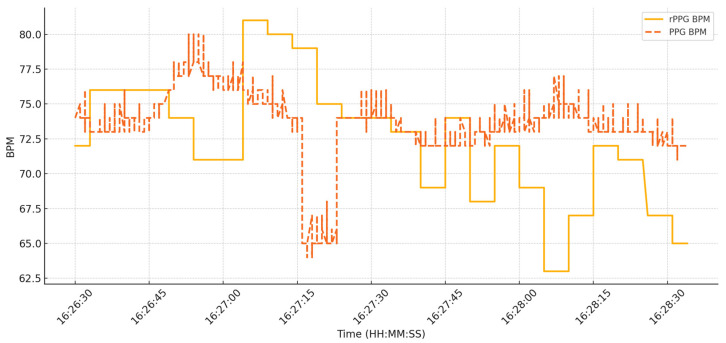
RPPG vs. PPG Sensor Comparison.

To evaluate the physiological feasibility of the smart mirror’s real-time MA-rPPG module, we conducted a single-subject comparative study using the Grove Ear-clip Heart Rate Sensor [44] connected to an Arduino Uno as the reference contact PPG system. This preliminary test illustrates feasibility rather than generalizable accuracy. Figure 8 presents the comparison between real-time rPPG and reference PPG BPM signals from this subject. The Pearson correlation coefficient (R) between the two signals was −0.093, with a Mean Absolute Error (MAE) of 4.17 BPM. Bland–Altman analysis showed a mean bias of −0.99 BPM, with 95% Limits of Agreement (LoA) ranging from −11.30 to +9.31 BPM. These single-subject results suggest that while the two methods differ, the feasibility of extracting pulse information from webcam-based rPPG in real time is demonstrated. However, variability can arise from environmental conditions such as lighting, camera angle, and facial reflectivity. With improved algorithms and noise compensation, non-contact rPPG remains a viable and scalable approach for health monitoring.

While the proposed system demonstrates the feasibility of using MA-rPPG for contactless heart rate estimation, the current results reveal weak agreement with the reference PPG sensor (R = −0.093, MAE = 4.17 BPM). This suboptimal performance can be attributed to several factors: (i) the use of a consumer-grade webcam without device-specific calibration; (ii) motion artifacts caused by natural head movements; (iii) lighting variations during data acquisition; and (iv) the very limited sample size (single-subject evaluation). Each of these factors is known to reduce signal-to-noise ratio and temporal alignment in rPPG signals, as also discussed in the recent literature on camera-based vital sign estimation. These results highlight the inherent difficulty of reliable physiological monitoring using non-contact methods under unconstrained conditions.

In future work, we plan to address these limitations by conducting experiments with a larger and more diverse participant group, implementing improved motion compensation and signal quality assessment techniques, and performing benchmarking against branded smartwatch with heart rate measurement, certified clinical-grade pulse oximeters or ECG-based heart rate monitors. Accordingly, the current physiological findings should be interpreted only as feasibility-stage evidence rather than claims of diagnostic capability or clinical equivalence. Such validation will be critical to establish the robustness and reliability of rPPG-based vital sign estimation for practical healthcare applications.

### 4.4. Affective Detection Accuracy

To enhance the interpretability and reliability of affective estimation, the smart mirror system focuses exclusively on real-time facial emotion recognition as an input for contextual feedback. Emotion detection is performed using the DeepFace library, which has demonstrated high accuracy in classifying basic emotional states such as happiness, sadness, anger, fear, and neutrality in real-time webcam conditions.

**Figure 9:**
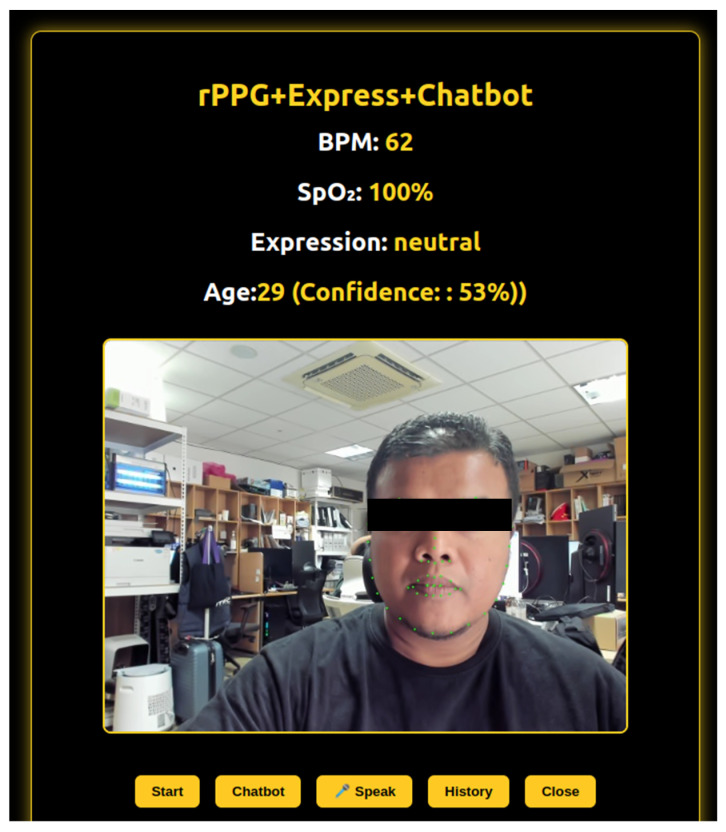
Confidence percentage for age.

By incorporating confidence scoring for age detection where the results collected for 30 times then take most frequent as final result, the system promotes cautious interpretation of AI-generated outputs and enhances user trust. These emotion predictions are used as part of the adaptive chatbot responses to provide contextually relevant wellness feedback. Figure 9 illustrates an example of how the detected emotion, and its confidence are displayed in the user interface.

### 4.5. Adaptive Chatbot Response Evaluation

To explore the contextual intelligence of the smart mirror’s integrated mental health assistant, we conducted a small formative assessment with a limited number of user interactions in a controlled office setting. Participants were instructed to display basic emotions (happy, neutral, sad, surprise) and engage in short chatbot conversations (see Table 2). The chatbot was queried using a fixed prompt structure that included real-time estimates of heart rate (BPM), blood oxygen saturation (SpO_2_), emotional state, and age. The goal was to examine system feasibility and functionality, not to establish generalizable accuracy or clinical efficacy. Performance was recorded in terms of latency, response relevance, and perceived empathy on a 5-point scale, and the results should therefore be interpreted as illustrative demonstrations of feasibility only. Below is the prompt:
“You are a chatbot integrated into a smart mirror system. This smart mirror uses a camera to extract real-time estimates of the user’s age, emotion, heart rate, and oxygen saturation. These values are AI-based estimations and may not always be accurate—please consider this when responding. Provide a short health and wellness summary (max 3 sentences), identify possible signs of stress, fatigue, or health concerns, and offer a simple lifestyle or recovery suggestion.”“User profile: Age: ${*age*}, Emotion: ${*expression*}, Heart Rate: ${*bpm*} BPM, SpO_2_: ${*spo2*}%.”

Across all profiles, the chatbot demonstrated sensitivity to emotional tone and physiological ranges. When stress-indicative metrics were detected—such as a high BPM (>100) coupled with emotions like fear or anger, the responses included suggestions for calming strategies. Conversely, when metrics fell within normal ranges and the emotion was neutral or positive, the responses affirmed wellness.

The GPT-4o model consistently generated empathetic, concise, and safety-aware recommendations, aligning with the system prompt constraints. It also refrained from clinical overreach, often using soft guidance language such as “consider,” “you may,” and “ensure.” Importantly, it respected the uncertainty in AI-generated data by avoiding deterministic statements.

**Figure 10:**
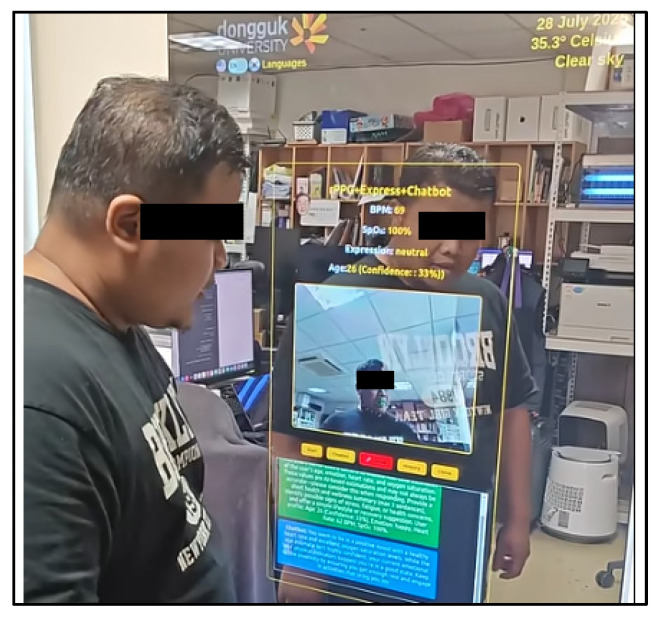
Screenshot of video demonstration.

To further enhance the understanding and novelty of our project, we have created a video demonstration that shows the interaction between user and the Smart Mirror (Figure 10). The video demonstration link is at Appendix A. The details pseudo-code provided in Appendix B and complete list of acronyms is summarized in Appendix C.

### 4.6. Functional Evaluation Across Emotion-Specific User Interactions

A total of 10 participants were involved in the experimental evaluation of the developed smart mirror system. The cohort represented a diverse set of national backgrounds, including individuals from Germany (*n* = 1), Uzbekistan (*n* = 2), Pakistan (*n* = 1), South Korea (*n* = 3), Malaysia (*n* = 2), and the Philippines (*n* = 1). The gender distribution comprised eight men and two women. This demographic diversity was intentionally included to ensure a broader representation of emotional responses. To assess the smart mirror chatbot’s ability to adaptively respond to emotion-specific states, a structured evaluation was conducted across five emotion prompts (happy, sad, neutral, fear, angry) for each of the 10 participants. Responses were rated on a 1–5 scale (1 worst, 5 best) across three key dimensions: relevance to physiological and emotional inputs, emotional empathy, and the quality of lifestyle suggestions. As shown in Table 3, the chatbot consistently demonstrated high relevance and empathy scores (mean scores exceeding 4.0), particularly for positive and negative emotional states such as happy and sad. However, slightly lower scores were observed in the “suggestion quality” metric for neutral and angry emotions, reflecting a tendency toward repetitive or generalized advice (e.g., “take a short walk” or “practice breathing”). These findings suggest that while the GPT-4o-powered chatbot can generate contextually appropriate responses, further prompt optimization or model fine-tuning may be necessary to enhance variation and emotional nuance, especially in less distinct affective contexts.

To investigate the variation and repetitiveness of chatbot-generated suggestions during real-time multimodal interactions, we analyzed the output logs for each participant across five emotion states. Table 4 summarizes a post hoc content analysis that categorized the chatbot’s wellness suggestions into key thematic patterns. While the GPT-4o-based assistant demonstrated strong contextual sensitivity to physiological and emotional cues, it also exhibited a tendency to repeat high-frequency advice such as “take a deep breath,” “go for a walk,” or “talk to someone,” especially in neutral, sad, or angry states. This repetition, although clinically safe and empathetic, highlights the current limitations in generative diversity when constrained by safety and brevity.

Although the chatbot generated contextually relevant and empathetic responses, the evaluation was limited to 10 participants (8 male, 2 female), restricting generalizability. The analysis also revealed repetitive wellness suggestions, indicating the need for improved prompt design or fine-tuning to enhance response diversity and personalization. Future work will address these limitations through larger and more gender-balanced user studies and by implementing adaptive prompt engineering strategies. 

## 5. Discussion

This study demonstrates the feasibility of transforming the pretrained MA-rPPG model—originally designed for offline analysis—into a real-time physiological sensing system embedded in an interactive smart mirror. The system integrates biometric signal processing, affective computing, and conversational AI into a seamless user experience. While encouraging, these findings are preliminary, being based on a single-subject physiological validation and a small formative user evaluation. They therefore provide feasibility evidence of technical integration and potential. Importantly, these results do not establish clinical efficacy, clinical-grade accuracy, or diagnostic capability, and all physiological metrics (e.g., heart rate, SpO_2_) should be regarded as preliminary prototype outputs pending rigorous multi-participant validation.

Ethical and privacy considerations are central to the design of the proposed system. The current prototype processes all biometric data locally, does not retain personal information, and was tested only with informed consent from volunteers. Nevertheless, we acknowledge known limitations in facial emotion recognition models, which may exhibit demographic bias across age, gender, and skin tone groups. Our evaluation cohort was small and imbalanced, further restricting generalizability. To mitigate these issues, future work will include larger and more diverse participant groups, bias-aware benchmarking, and incorporation of fairness-enhancing techniques in model training and evaluation.

### 5.1. Innovations in Real-Time Deployment

The proposed system focuses on engineering contributions in real-time deployment and integration, leveraging existing state-of-the-art AI models with proven benchmarks rather than retraining or developing new architectures. To realize real-time interaction, we re-engineered the MA-rPPG pipeline into a persistent, low-latency backend system optimized for continuous webcam streaming. Key innovations in this deployment include:

- Persistent GPU Threading for MA-rPPG: The backend initiates all core MA-rPPG model components (generator, keypoint detector, head pose estimator) within a continuous GPU-based worker thread (gpu_worker), avoiding repeated model instantiation and reducing latency.
- Live Video Capture and Processing: A dedicated frame acquisition thread (webcam_worker) captures facial input, performs live face detection, and feeds data directly into the inference pipeline, enabling responsive, frame-wise analysis.
- Dynamic Signal Estimation: The pipeline incorporates advanced filtering (0.7–3.5 Hz bandpass) and peak detection to extract heart rate and SpO_2_ data accurately from live video, addressing the challenges of motion artifacts and signal noise.
- Multimodal AI Fusion: Real-time facial expressions, demographic attributes, and physiological metrics are fused into a contextual summary. This triggers an interactive feedback loop via a GPT-4o-based mental health chatbot, with responses rendered both textually and audibly through text-to-speech synthesis.

The following Table 5 succinctly highlights the contributions and advancements of our real-time smart mirror deployment compared to the original offline MA-rPPG methodology:

### 5.2. Comparative Analysis of Existing Smart Mirror Systems

To contextualize our contribution, Table 6 compares our system with prominent smart mirror solutions from recent literature. While prior works have explored individual modalities (emotion recognition, sentiment analysis, or rPPG), our platform is the first to unify all three—physiological sensing, emotional inference, and conversational AI—within a real-time, bilingual smart mirror system.

### 5.3. Design Considerations for Ambient and Human-Centered Use

Beyond its technical architecture, the design of the proposed smart mirror system prioritizes human-centered interaction in ambient settings, with particular emphasis on accessibility, intuitiveness, and cultural adaptability. This design philosophy aligns closely with the goals of Ambient Assisted Living (AAL) systems, which aim to support users—particularly the elderly and non-technical individuals—through non-intrusive, context-aware digital interventions.

First, the graphical user interface (GUI) has been developed with a strong focus on simplicity and clarity. Its minimalist layout, large text elements, and high-contrast visual cues are intended to reduce cognitive load, making it easier for users with vision impairment, reduced motor skills, or limited digital literacy to interact with the system confidently. The bilingual interface, which supports both Korean and English with a simple toggle mechanism, reflects an understanding of language inclusivity and the multilingual nature of many real-world user environments.

Second, the decision to embed the system within a two-way acrylic mirror enables a naturally intuitive user experience. Unlike wearable devices or traditional medical monitoring equipment that require conscious use or bodily attachment, the smart mirror invites passive interaction. Users receive personalized health insights simply by looking into the mirror, with no need to initiate commands or navigate complex menus—an important feature for encouraging daily use, especially among elderly users or those managing cognitive decline.

Third, the integration of emotion recognition and physiological sensing into the AI chatbot interaction loop allows for empathetic, personalized feedback tailored to the user’s current mental and physical state. This context-aware design ensures that interactions are not only functional but also emotionally resonant, which is critical for user trust, engagement, and long-term adherence in mental health applications.

Fourth, the system’s architecture is built for real-world viability. All processing occurs locally on an edge device to maintain user privacy and reduce dependency on external cloud services, which is often a concern in sensitive health-related deployments. The modular software design also allows future customization for users with different needs, such as children, caregivers, or patients with specific medical conditions.

Fifth, a key limitation of this study is the absence of comparative benchmarking against alternative models or clinical-grade devices. This is due to limited computational resources, financial constraints, and the lack of access to clinical collaborators or participant cohorts for real-world testing. Therefore, we relied on models that have already been validated in prior research which are state-of-the-art AI models (MA-rPPG [11], DeepFace [12], and GPT-4o [18,19,20,21]). Future work will focus on establishing collaborations with clinical institutions to perform user studies and comparative evaluations.

Lastly, while our chatbot generated context-aware responses based on real-time physiological and emotional inputs, this system should be interpreted as an awareness and self-reflection tool rather than a clinical mental health assessment platform. Our evaluation demonstrates preliminary feasibility of integrating validated AI modules for real-time multimodal interaction. However, it is important to note that the physiological validation was limited to a single subject, and the user evaluation constituted only a small formative assessment. Therefore, the findings should not be interpreted as evidence of clinical effectiveness or generalizable accuracy. Future work will involve collaborations with mental health professionals to conduct larger-scale evaluations and to assess user experience, perceived support, and long-term adherence.

Taken together, these design choices establish the smart mirror as a scalable, unobtrusive, and human-friendly interface for multimodal health monitoring—positioning it not just as a technological artifact, but as a meaningful contributor to ambient digital healthcare.

## 6. Conclusions and Future Work

In conclusion, the integration of multimodal AI methodologies, including MA-rPPG-based vital sign estimation, DeepFace-driven facial attribute analysis, and conversational AI, enabled the development of a feasibility-stage smart mirror platform for ambient health monitoring. The system demonstrates preliminary effectiveness in the real-time extraction of physiological and emotional parameters, allowing personalized, non-intrusive user interactions. However, these findings are based on single-subject physiological validation and a small formative user evaluation and thus should be interpreted strictly as feasibility evidence of technical integration rather than clinical efficacy. Although the proposed smart mirror successfully integrates multimodal sensing with conversational AI, its current scope is limited to real-time contextual feedback for general wellness. The system does not store user data or perform longitudinal tracking, and it is not intended to replace professional clinical evaluation. Future research will include user studies and collaborations with healthcare institutions to validate its role in mental health support. It is important to emphasize that the current study should be regarded as an early-stage feasibility demonstration of real-time multimodal interaction and health awareness, not as a clinically validated monitoring solution. The system’s physiological sensing accuracy remains limited, primarily due to single-subject evaluation, the absence of calibration, and the lack of benchmarking against medical-grade devices. Future research will therefore focus on conducting larger user studies in collaboration with healthcare institutions and employing certified pulse oximeters and ECG reference devices to quantitatively validate heart rate and SpO_2_ estimation performance. These efforts will ensure that subsequent versions of the smart mirror can achieve the level of reliability required for clinical or remote patient monitoring scenarios. Additionally, future work will explore advanced lightweight deep-learning models, optimized edge-computing techniques, and integration with other assistive technologies (e.g., service robots) to further enhance the mirror’s responsiveness and practicality for broader healthcare applications.

## Figures and Tables

**Table 1:** GPU Usage and Latency Performance Metrics.

Module	GPU Usage (%)	Latency (ms)	Response Time (ms)
Real-time rPPG	18–22%	~160–210 ms	
Real-time SpO_2_	14–18%	~180–240 ms	
Deepface (Emotion)	25–30%	~350–450 ms	
Deepface (Age)	28–32%	~500–600 ms	
Chatbot API Response	Idle		~1200–1800 ms

**Table 2:** Test Cases and Chatbot Responses.

Case	Input Profile	GPT-4o Response Summary
1	Age: 25, Emotion: happy, HR: 78 BPM, SpO_2_: 98%	“You appear to be in good physical and emotional health. No immediate signs of stress detected. Keep up your current routine and stay hydrated.”
2	Age: 42, Emotion: angry, HR: 105 BPM, SpO_2_: 94%	“Elevated heart rate and emotional tension suggest possible stress. Consider taking deep breaths or a short walk. Try to relax and reduce any triggers.”
3	Age: 67, Emotion: neutral, HR: 85 BPM, SpO_2_: 89%	“Your oxygen levels are slightly low. You may be feeling mild fatigue. Ensure proper ventilation and consider medical advice if symptoms persist.”
4	Age: 30, Emotion: sad, HR: 72 BPM, SpO_2_: 97%	“You may be feeling emotionally low. It’s okay to take a break and talk to someone you trust. A short walk or connecting with a friend could help.”
5	Age: 52, Emotion: fearful, HR: 112 BPM, SpO_2_: 92%	“High heart rate and fear may indicate anxiety or acute stress. Practice calming techniques like controlled breathing. You’re not alone, consider reaching out.”

**Table 3:** Chatbot Response Evaluation.

User	Emotion (Happy|Sad|Neutral|Fear|Angry)		
	Relevance (1~5)	Empathy (1~5)	Suggestion (1~5)
User 1	5|5|5|5|4	5|5|5|5|4	5|5|5|5|3
User 2	5|5|5|5|5	5|5|5|5|5	5|5|5|5|5
User 3	4|5|5|5|5	3|5|5|5|5	4|5|5|5|5
User 4	4|5|5|5|5	3|5|5|5|5	4|5|5|5|5
User 5	5|4|5|5|5	5|3|5|5|5	5|4|5|5|5
User 6	4|5|5|5|5	3|5|5|5|5	3|5|5|5|5
User 7	4|4|4|5|3	3|3|4|5|4	4|3|3|5|4
User 8	5|5|5|5|5	5|5|5|5|5	5|5|5|5|5
User 9	5|5|5|5|5	5|5|5|5|5	5|5|5|5|5
User 10	5|5|5|5|5	5|5|5|5|5	5|5|5|5|5

**Table 4:** Repeated Chatbot Suggestion Analysis.

Suggestion Phrase	Frequency
Short Walk	19
Mindfulness	17
Deep breathing	15
Practice breathing	6
Meditation	4
Short break	3
Relaxing Activity	3
Step outside	3

**Table 5:** Comparison between original Ma-rPPG and our near real-time approach.

Features	Original Ma-rPPG	Our Approach
Processing Mode	Batch (Offline)	Real-time continuous (streaming)
Input Source	Pre-recorded video	Live Webcam feed
Latency	High (post-processing)	Low (immediate output)
Inference Engine	Single-run inference	Persistent, threaded GPU inference
Physiological Metrics	Heart Rate (static)	Heart Rate (dynamic real-time)
Deployment	Laboratory	User-interactive smart mirror
User Experience	Non-interactive	Interactive, continuous engagement

**Table 6:** Comparative Summary of Smart Mirror Systems.

References	Modalities	Real-Time	Emotion Detection	AI Chatbot	Strength and Limitation
Bianco et al. (2021) [3]	Emotion Monitoring	Yes	Yes	No	Facial and vocal emotion sensing but no physiological sensing or AI feedback
Yu et al. (2021) [4]	Sentiment Analysis	Yes	Yes	No	Korean-language sentiment model but no physiological or multimodal fusion
Chaparro et al. (2021) [8]	Ambient Assisted Living (AAL), Rehab Support	Partial	No	No	Elder-focused rehab tools but no real-time emotion or chatbot integration
Casalino et al. (2025) [9]	rPPG (HR, SpO_2_)	Yes	No	No	Low-cost real-time rPPG mirror but no mental health or affective feedback
Proposed System	rPPG + Emotion + Chatbot	Yes	Yes	Yes	Full-stack integration of real-time physiological and affective sensing with AI chatbot feedback

## Data Availability

The simplified implementation of the smart mirror backend (GPU worker for MA-rPPG, DeepFace, and OpenAI’s GPT-4o model (May 2025 release) accessed via RESTful API) is available at: https://github.com/mafifkasno/realtime_ma-rppg_emotion_chatbot_smartmirror (accessed on 16 September 2025).

## Appendix A. Video Demonstration

A video demonstration of the AI Multimodal Smart Mirror as below: https://www.youtube.com/shorts/-lAFdtdqsoI?feature=share (accessed on 16 September 2025).

## Appendix B. Pseudo-Code of Interfacing Implementation

For enhanced readability, the pseudo-code clearly separates backend and frontend workflows into structured, descriptive procedures, facilitating better understanding of the modular interactions and data flows.

**Algorithm A1** Backend Processing Pipeline for Integrated rPPG, Facial Analysis, and Chatbot Interaction
**Input:** Webcam video stream **Output:** Real-time BPM, SpO_2_, Facial Attributes (Emotion, Age **Initialize:** Load AI models: MA-rPPG, DeepFace Initialize queues **and** shared variables **for** inter-process communication Start Flask API server **Procedure MAIN():** Start threads: THREAD WebcamWorker() THREAD GPUWorker() **Procedure WebcamWorker():** **while not** StopSignal: Capture frame **from** webcam Detect face **and** landmarks using dlib Annotate landmarks on frame Enqueue annotated frames into FrameQueue Sleep **for** frame interval (30 fps) **Procedure GPUWorker():** Initialize buffers: bpm_buffer, spo2_buffer Initialize counters: frame_counter = 0 ** while not** StopSignal: Dequeue frame **from** FrameQueue Preprocess frame **for** model inference frame_counter += 1 **if** (frame_counter mod 5 == 0): Estimate BPM using MA-rPPG model: Generate synthetic animation Calculate rPPG signals Apply bandpass filter Detect peaks Compute BPM Update BPM shared variable **if** (frame_counter mod 15 == 0): Estimate SpO_2_ **from** recent ROI frames: Extract RGB channel means Calculate AC/DC ratios Compute SpO_2_ Update SpO_2_ shared variable Perform facial analysis **with** DeepFace every frame until thresholds are met: **if** Age estimation incomplete: Analyze age Accumulate results (30 samples) Update shared age result **with** highest frequency **if** (frame_counter mod 30 == 0): Analyze emotion using DeepFace Update shared emotion result Procedure FlaskAPI(): Route/start: Initialize webcam capture Clear stop signal Start MAIN() Route/stop: Set stop signal Release webcam Clear GPU memory Reset all shared variables **and** buffers Route/result: Return JSON containing: - BPM - SpO_2_ - Emotion - Age (**with** confidence) Route/video_feed: Stream JPEG frames **from** processed output queue Run FlaskAPI at host address (e.g., http://localhost:5004)

**Algorithm A2** Frontend Interaction Pipeline for Smart Mirror Interface
**Input:** Real-time data **from** Backend API **Output:** GUI Display **with** Real-time Vital Signs, Facial Attributes, and Chatbot Interaction **Initialize:** Set state variables: BPM, SpO_2_, Emotion, Age Initialize video feed URL Initialize messages queue **for** chatbot Initialize status indicators **Procedure MAIN():** Display GUI: - Vital signs (BPM, SpO_2_) - Facial attributes (Emotion, Age) - Live webcam feed - Chat history - Interactive buttons (Start, Chatbot, Speak, Stop) **Procedure handleStart():** Send POST request to backend API to initiate processing Set video feed URL to backend webcam stream Start polling backend API for results at regular intervals (every 1 second) **Procedure pollResult():** Fetch results **from** backend API: - BPM - SpO_2_ - Emotion - Age **with** confidence Update state variables accordingly Check **if** all data (BPM, SpO_2_, Emotion, Age) are valid: **if** valid: Set dataReady = true **else:** Set dataReady = false **Procedure handleChatbot():** **if not** dataReady: **return** Construct chatbot prompt **with** current state values: - Age, Emotion, BPM, SpO_2_ Send chatbot prompt to backend TTS API Receive chatbot response text **and** audio URL Display chatbot response **in** GUI chat history Play audio response **from** chatbot **Procedure handleSpeak():** Activate browser-based speech recognition: Set language based on user preference (English/Korean) Capture speech input **and** convert to text Send captured text to chatbot backend API Receive **and** display chatbot textual **and** audio response **Procedure handleStop():** Send request to backend to stop data acquisition Stop polling **for** results Clear video feed Reset all state variables Close application interface **Display** Status Messages: **if** any errors encountered during interactions: Display user-friendly error message in GUI Run MAIN to render **and** manage real-time GUI interactions

## Appendix C. List of Acronyms

**Acronym**	**Full Form**	**Description**
AI	Artificial Intelligence	Computer systems are designed to perform tasks that normally require human intelligence, such as learning and reasoning.
rPPG	Remote Photoplethysmography	A non-contact technique to estimate heart rate and other vital signs using video-based analysis of subtle skin color changes.
MA-rPPG	Moving Average Remote Photoplethysmography	An enhanced rPPG method that uses motion-augmented training for more robust vital sign estimation.
SpO_2_	Peripheral Oxygen Saturation	A measure of the amount of oxygen-carrying hemoglobin in the blood relative to the amount of hemoglobin not carrying oxygen.
PPG	Photoplethysmography	A contact-based optical technique to measure blood volume changes in the microvascular bed of tissue.
FER	Facial Emotion Recognition	AI-based analysis of human emotions from facial expressions.
CNN	Convolutional Neural Network	A deep learning model commonly used for analyzing visual data such as images and videos.
ROI	Region of Interest	A selected area in an image or video frame used for focused analysis or processing.
GUI	Graphical User Interface	The visual part of a software application that allows users to interact with the system through graphical elements.
API	Application Programming Interface	A set of rules and endpoints that enable software applications to communicate with each other.
BPM	Beats Per Minute	A unit used to measure heart rate or pulse.
MAE	Mean Absolute Error	A metric used to measure the average magnitude of errors between predicted and true values.
LoA	Limits of Agreement	Statistical measure that defines the range within which most differences between two measurement methods lie.
GPT-4o	Generative Pre-trained Transformer 4o	An advanced large language model developed by OpenAI, used for generating text and conversational AI responses.
TTS	Text-to-Speech	Technology that converts written text into spoken voice output.
STT	Speech-to-Text	Technology that converts spoken language into written text.
CBT	Cognitive Behavioral Therapy	A psychological treatment method that helps patients manage problems by changing patterns of thinking or behavior.
